# *Agrobacterium*-Mediated Transformation of Russian Commercial Plum cv. “Startovaya” (*Prunus domestica* L.) With Virus-Derived Hairpin RNA Construct Confers Durable Resistance to PPV Infection in Mature Plants

**DOI:** 10.3389/fpls.2019.00286

**Published:** 2019-03-12

**Authors:** Tatiana Sidorova, Roman Mikhailov, Alexander Pushin, Dmitry Miroshnichenko, Sergey Dolgov

**Affiliations:** ^1^Branch of Shemyakin-Ovchinnikov Institute of Bioorganic Chemistry, Russian Academy of Sciences, Puschino, Russia; ^2^Nikita Botanical Gardens – National Scientific Centre, Russian Academy of Sciences, Yalta, Russia; ^3^All-Russia Research Institute of Agricultural Biotechnology, Russian Academy of Science, Moscow, Russia

**Keywords:** *Plum pox virus*, European plum, stone fruits, genetic transformation, leaf explant, RNA interference

## Abstract

In modern horticulture *Plum pox virus* (PPV) imposes serious threats to commercial plantations of a wide range of fruit species belonging to genera *Prunus*. Given the lack of natural genetic resources, which display reliable resistance to PPV infection, there has been considerable interest in using genetic engineering methods for targeted genome modification of stone fruit trees to control Sharka disease caused by PPV. Among the many virus defense mechanisms, RNA interference is shown to be the most promising transgenic disease-control strategy in plant biotechnology. The present study describes the production of transgenic PPV resistant European plum “Startovaya” (*P. domestica* L.) through the *Agrobacterium*-mediated transformation of *in vitro* leaf explants. Due to organogenesis from leaves, the established protocol allows the genetic engineering of the plum genome without losing clonal fidelity of original cultivar. Seven independent transgenic plum lines containing the self-complementary fragments of PPV-CP gene sequence separated by a PDK intron were generated using *hpt* as a selective gene and *uidA* as a reporter gene. The transformation was verified through the histochemical staining for β-glucuronidase activity, PCR amplification of appropriate vector products from isolated genomic DNA and Southern blot analysis of hairpin PPV-CP gene fragments. To clarify the virus resistance, plum buds infected by PPV-M strain were grafted onto 1-year-old transgenic plants, which further were grown into mature trees in the greenhouse. As evaluated by RT-PCR, DAS-ELISA, Western blot, ImmunoStrip test, and visual observations, GM plum trees remained uninfected over 9 years. Infected branches that developed from grafted buds displayed obvious symptoms of Sharka disease over the years and maintained the high level of virus accumulation, whereby host transgenic trees had been constantly challenged with the pathogen. Since the virus was unable to spread to transgenic tissues, the stable expression of PPV-derived gene construct encoding intron-spliced hairpin RNAs provided a highly effective protection of plum trees against permanent viral infection. At the same time, this observation indicates the lack of the systemic spread of resistance from GM tissues to an infected plum graft even after years of joint growth.

## Introduction

The world production of stones fruits has long been suffered from Sharka disease. For a century this quarantine disease, caused by a linear single-stranded RNA *Plum pox virus* (PPV), negatively affect the yield and quality of plum (*Prunus domestica*), apricot (*Prunus armeniaca*), peach (*Prunus persica*), Japanese plum (*Prunus salicina*), and cherry (*Prunus avium*) in various horticultural areas of Europe, Asia, and North Africa (Scholthof et al., 2011; Garcia et al., 2014; Rimbaud et al., 2015). Since the traditional chemical and biological control practices are not effective for recovering infected plants, the best way to control the disease in stones fruits orchards is to use the resistant genotypes.

Despite the many years of traditional intra- and interspecies crosses within *Prunus* genera there is still no great success in producing virus-resistant varieties. The limited result of conventional breeding for Sharka disease resistance is not only due to the extended generation time of fruit trees and incompatibility barriers but also because of the restricted number of available sources displaying longtime resistance to PPV. Due to an uncharacterized genetic control of PPV resistance in discovered natural sources, the conventional breeding is highly unpredicted and requires a longtime evaluation of seedlings for viral resistance.

In plum (*P. domestica*), the natural host of the PPV, the most known sources for breeding are hypersensitive cultivars, such as “Jojo,” K4-Hybride, Ort × Stan 34, which are able to withstand for years to natural virus infection (Neumüller and Hartmann, 2008). Young trees or budsticks of such cultivars, however, frequently died off within a few weeks due to hypersensitivity to PPV caused by grafting onto virus-infected rootstocks. The death of the entire tree can also occur in the case of natural aphid-mediated inoculation when a susceptible rootstock becomes systemically infected before the hypersensitive reaction in a scion (Rimbaud et al., 2015). Since only a full-grown tree can safely resist both natural and artificial PPV infection, the rigorous controlling for the latent infection in nurseries and young orchards is required to prevent massive loss of the trees. Since the grafting of various scion/rootstock combinations is the widespread practice in the stone fruits industry, the hypersensitive germplasms are less preferable candidates for commercial orchards, than the fully resistant cultivars.

Over the past 30 years, the full resistance to PPV has been developed in plum by genetic engineering. The success of the transgenic disease-control strategy is based on the specific degradation of viral RNA through the mechanism of RNA interference. Historically, “HoneySweet” (originally C-5) is the first and still the most known PPV resistant transgenic European plum, which was produced in the early 90s before the RNA interference become a widespread biotechnology (Scorza et al., 1994). At that period several research groups tried to achieve pathogen resistance in model plants and *Prunus* species by the post-transcriptional gene silencing, introducing constructs that express antisense or sense sequences derived from various segments of the PPV genome. Most of those viral-protein-mediated investigations failed to obtain reliable resistance in transgenic plants (reviewed by Ilardi and Tavazza, 2015; Limera et al., 2017). Fortunately, C-5 has generated after the spontaneous rearrangement of PPV coat protein (CP) sequences into an inverted repeat/hairpin configuration during the *Agrobacterium*-mediated transformation. This resulted in activation of RNA silencing process in regenerated C-5 plant (Kundu et al., 2008), and thus provided long time viral resistance confirmed later by various greenhouse tests and field trials (Capote et al., 2008; Polák et al., 2008, 2017; Wong et al., 2009; Scorza et al., 2013). Soon it was shown that the introduction of ihpRNA constructs, specifically engineered by joining of the viral fragment as inverted repeats separated by a spacer or an intron, significantly increase the silencing efficiency in host plants (Khalid et al., 2017). Important to note that in contrast to vectors designed to express viral genes in the plant genome, hairpin-shaped sequences avoid the accumulation of transgene-derived proteins in plant cells. The successful introduction of the ihpRNA constructs into the plum genome via genetic transformation was first reported in 2007 (Hily et al., 2007). Due to the constitutive expression of an intron-spliced sequence of the full PPV CP, greenhouse-grown plum plants showed the systemic resistance to various PPV isolates. Soon after, the ihpRNA construct containing the part of the 5′ sequences of the PPV *P1* gene was also reported to provide the viral resistance to Canadian PPV-D strain in transgenic plum (Wang et al., 2009). Later, the hairpin vector expressing PPV-M derived *P1* sequences has been successfully used to induce the resistance to an Italian PPV-M strain in two transgenic plum events (Monticelli et al., 2012). In the past few years, new modifications of ihpRNA constructs, encoding fragments or full sequences of *P1* or *CP* PPV genes were introduced by various groups into European plum genome by genetic engineering (Petri et al., 2011; Wang et al., 2013; García-Almodóvar et al., 2015).

In recent years, intensive studies on the interaction of eukaryotic translation initiation factors with viral proteins provided a new insight of PPV resistance mechanism in plants (Decroocq et al., 2006; Marandel et al., 2009; Wang and Krishnaswamy, 2012). As a result, the ihpRNA-mediated knockdown of the specific isoform of translation initiation factor eIF4 provided a notable resistance to PPV in transgenic *P. domestica* (Wang X. et al., 2013). Taking into account the fast development of the plant genome editing techniques, the targeted mutation of host genes involved into the replication and wide-spreading of PPV in infected tissues would be a very promising approach for engineering virus resistance that excludes the introduction of foreign sequences into the plum genome.

Unfortunately, the implementation of various virus-defense approaches in plum is hampered by its high recalcitrance for the *in vitro* organogenesis. In fact, the listed above transgenic plum events are the results of genetic modification of seed-derived tissues. Therefore, all generated PPV-resistant transgenic plum lines do not match the original characteristic of the genotypes “Bluebyrd,” “Stainley,” and “Claudia Verde” used as sources for genetic transformation. Since plants were regenerated from embryonic cotyledons or hypocotyls, they are representing a new genetic mix of the parent genome. No doubt, the popular transformation protocols optimized for plum embryonic tissues are rather effective in producing transgenic plants with a frequency up to 42% (Petri et al., 2008; Tian et al., 2009; Srinivasan et al., 2012; Faize et al., 2013), but the heterogeneity of regenerated plants is a serious problem with their direct commercial use.

The original agronomic characteristics of genotype could be preserved when genome modification will be followed by the regeneration from somatic tissues, such as leaves, petioles, stem segments, roots, or meristems. In plum, the number of reports describing the successful regeneration from somatic explants is particularly limited (Escalettes et al., 1994; Yancheva et al., 2002; Petri and Scorza, 2010). In our laboratory, we have developed an efficient protocol for shoot regeneration from leaf explants of *in vitro* propagated plum shoots (Mikhailov and Dolgov, 2007). It relies on the accurate choice of leaves at the optimal physiological age, the obligatory explant pretreatment in a liquid medium, and the proper combinations of plant growth regulators and additional supplements. On the basis of a leaf-mediated regeneration approach, two protocols for the *Agrobacterium*-mediated transformation of European plum “Startovaya” have been developed using the positive (*PMI*) and the negative (*hyg*) selection schemes (Mikhailov and Dolgov, 2007; Sidorova et al., 2017). Since the mid-2000s we have tried to produce PPV resistance in European plum “Startovaya” by the overexpression of viral-derived sequences in transgenic plants (Dolgov et al., 2010). Two approaches were chosen to generate PPV resistant plum; one was based on the co-suppression mechanism and another on RNA-silencing. Beginning from 2008 several rounds of grafting with infected by PPV-M buds were performed on transgenic plum plants. Our attempt to express the construct containing the PPV *CP* gene in sense-orientation driven by a strong promoter was not successful for protecting transgenic “Startovaya” plants from the virus (Dolgov et al., 2010). In contrast, the preliminary experiment with the PPV CP derived gene construct encoding intron-spliced hairpin RNAs showed a promising result since no viral symptoms were found (Dolgov et al., 2010).

Here we present the results our decennial efforts directed to produce PPV resistant plum plants from somatic tissues, starting from the *Agrobacterium*-mediated transformation experiments aimed to generate primary transgenic shoots and ending with the results of 9-year greenhouse evaluation for PPV resistance of transgenic plum trees after the artificial virus delivery by graft inoculation.

## Materials and Methods

### Plant Material

Russian commercial cultivar plum “Startovaya” (*P. domestica* L.)^1^ was used in the study. *In vitro* shoots of “Startovaya” were multiplied on proliferation medium consisted of JS macro- and micro-elements (Jacoboni and Standardi, 1982) and MS vitamins (Murashige and Skoog, 1962), 2% sucrose (w/v), 0.7% agar (w/v), 100 mg/L myo-inositol, 1.5 mg/L benzylaminopurine (BAP), 0.1 mg/L indole butyric acid (IBA), pH 5.8. For transformation experiments, the shoots were transferred to rooting medium (RM), which contained ½-strength JS salt and MS vitamins, 0.5 mg/L IBA, 0.7% (w/v) agar, pH 5.8. Rooted plants were used as donors for leaf explants. All cultures were grown at 24 ± 1°C under a 16/8-h photoperiod with light provided by an equal mixture of cool-white and Gro-Lux lamps.

### *Agrobacterium* Strain and Vector

The supervirulent *Agrobacterium* strain AGL0 (Lazo et al., 1991) were used for plum transformation. To produce PPV resistant plum the binary vector pCamPPVRNAi (Dolgov et al., 2010) was used. This vector contains the *hpt* gene under the duplicated cauliflower mosaic virus 35S promoter (d35S), the *uidA* gene under the CaMV 35S promoter and self-complementary fragments of PPV *CP* gene under the modified enh35S promoter, with a duplicated enhancer sequence. The *uidA* gene contains a 190 bp Castor bean catalase intron to prevent expression in bacterial cells. The fragments of the PPV *CP* gene were separated by a PDK intron (from pHANNIBAL) to produce a hairpin RNA (hpRNA) structure in antisense-sense orientation. *Agrobacterium* cultures were grown and prepared for plum transformation as described (Sidorova et al., 2017).

### Transformation and Selection of Putative Transgenic Plants

The 2–4 youngest fully expanded leaves of 5 to 10-week-old shoots were obtained from the *in vitro* rooted plants. Preparation of explants, pretreatment in auxin-reached liquid-medium and incubation with *Agrobacterium* were carried out as described (Sidorova et al., 2017). Incubated leaf explants were transferred onto the shoot regeneration medium consisted of JS salts and MS vitamins, 3% sucrose (w/v), 0.7% agar (w/v), 100 mg/L myo-inositol, 5 mg/L BAP, 0.5 mg/L IBA, 4.0 mg/L calcium pantothenate, pH 5.8., and co-cultivated for 3 days in the dark at 23°C. Following the co-cultivation step, explants were transferred onto the shoot regeneration medium supplemented with 500 mg/L cefotaxime to eliminate *Agrobacterium*. Cultures were maintained in the dark for 3 weeks and then were transferred to light with a 16-h photoperiod onto the fresh shoot regeneration medium supplemented with 500 mg/L cefotaxime and 5 mg/L hygromycin for the selection of transgenic morphogenic callus. The callus by degrees appeared 25–30 days after induction, and the decayed tissues were carefully removed. After 2–2.5 months from the beginning of transformation, morphogenic calli were transferred onto the shoot elongation medium, which consisted of JS salts and MS vitamins, 30 g/L sucrose, 7% agar (w/v), 100 mg/L myo-inositol, 2 mg/L BAP, 0.1 mg/L IBA, pH 5.8, and supplemented with 300 mg/L cefotaxime and 5 mg/L hygromycin. The morphogenic calli were sub-cultured at a frequency of 10 and 14 days. Shoots that survived after 2–4 months culture on the shoot elongation medium were excised from calli and propagated on the proliferation medium containing 5 mg/L hygromycin. When putative transgenic shoots reached 3 cm in length, they were transferred to rooting medium supplemented with 3 mg/L hygromycin. Five to seven weeks after the initial rooting plantlets were transferred to pots (14 cm × 10.5 cm) in the greenhouse.

### Analysis of Putative Transformants

#### Analysis of Reporter Genes Expression

In transient expression experiment, visual screening for GFP fluorescence was performed using ZEISS SteREO Discovery.V12 microscope equipped with PentaFluar S 120 vertical illuminator and filter sets 57 GFP BP (EX BP 470/40, BS FT 495, EM LP 550) (Carl Zeiss MicroImaging GmbH, Germany). The numbers of GFP foci were counted 1, 3, 6, 9, 12, 15, and 22 days after bacterial infection and the results were analyzed to determine the percentage of explants with GFP-expressing cells. Seventeen to twenty two explants were cultured in individual Petri dishes as one replicate. All transient expression assays were repeated at least three times.

The GUS staining of different tissues was carried out accordingly to Jefferson et al. (1987).

#### PCR Analysis and Southern Blot Hybridization

Genomic DNA was extracted from young leaves of plantlets which had been growing in the selection medium using the cetyltrimethylammonium bromide (CTAB) method as described (Rogers and Bendich, 1994). The presence of transferred sequences was confirmed by PCR analysis using gene-specific primers. To provide amplification of a 1476 bp fragment comprising the sequence of the modified CaMV 35S promoter and a part of the PPV *CP* the forward primer 35S712For (5′-CAGCAGGTCTCATCAAGACGATCTACC-3′) and the reverse primer PPVUpRNAi (5′-AAGAGAAGACCTGGAGGAAGTTGATG-3′) were used. For the amplification of an 897 bp fragment comprising the sequence of a part of PPV *CP* gene and the octopine synthase gene terminator the forward primer PPVUpRNAi (5′-AAGAGAAGACCTGGAGGAAGTTGATG-3′) and the reverse primer OcsTerRev (5′-AGTAGTAGGGTACAATCAGTAAATTGAACGGAG-3′) were used. The primer sequences for the β-glucuronidase gene were: forward, 5′-TCGTAATTATGCGGGCAACGTC-3′ and reverse, 5′-CGAATCCTTTGCCACGCAAG-3′; and *hpt* gene: forward, 5′-CGACGTCTGTCGAGAAGTTTCTGATC-3′ and reverse, 5′-GTACTTCTACACAGCCATCGGTCCA-3′, corresponding to a 740 and 951 fragment, respectively. The amplified DNA fragments were visualized under ultraviolet light after electrophoresis on 1.0% agarose gel containing a TAE running buffer and ethidium bromide.

Southern Blot analysis was performed as described (Sidorova et al., 2017) using an 897 bp PCR amplified fragment corresponding to the coding sequence of the PPV *CP* gene and the octopine synthase gene terminator as a probe.

#### Northern Blot Hybridization

An extracted plum total RNA (20 μg) was loaded onto 15% polyacrylamide gel containing 7 M urea, and then electrotransferred onto a membrane (Hybond-N+, GE Healthcare, United Kingdom). siRNA bands were probed within the PPV *CP* 897 bp PCR fragment that was labeled with alkaline phosphatase using the Amersham Gene Image AlkPhos Direct Labeling and Detection System (GE Healthcare, United Kingdom). Detection was performed using CDP-Star detection reagent following manufacturer’s directions (Amersham CDP-Star Detection reagent, GE Healthcare, United Kingdom).

### Grafting for Virus Inoculation

In March 2009, 1-year-old plants of five independent transgenic events, containing PPV-hpRNA construct and non-transgenic “Startovaja” plants were inoculated by budding with *Plum pox virus*, Marcus strain (PPV-M), isolate PS (AJ243957). The infected 1-year-old bud wood was obtained from Federal State Budget Scientific Institution «North Caucasian Federal Scientific Center of Horticulture, Viticulture, Wine-making» (Russia, Krasnodar). Virus inoculation was conducted through the grafting of an individual virus-containing bud onto the transgenic plant. For each independent transgenic line from 10 to 12 plants were inoculated. Following grafting, the plants were maintained in the greenhouse at 22–24°C/16–18°C (day/night) for 7 months at natural light, and then the plants went through a cold treatment (about 6°C) for 4 months. In the spring of 2010, plants with survived infected buds converted into branches were visually monitored for PPV symptoms after cold treatment. Plants with died off buds were discarded from the assessment.

### Analysis of Infected Plants

The grafted plants/trees were grown in a greenhouse and evaluated every vegetative season for visual symptoms of virus infection. To verify the presence PPV in the leaves of transgenic and WT plants the molecular analysis was performed in 2010, 2014, and 2018. In spring 2010 viral infection was first monitored through visual symptoms, followed by RT-PCR analyses for the presence of PPV *HC-Pro* and 3′UTR sequences. In 2014 Double Antibody Sandwich ELISA (DAS-ELISA), ImmunoStrip test (Agdia, United States), Western blot analysis and amplification of *HC-Pro* gene and RNA-dependent RNA polymerase gene of virus genome were used. In the spring of 2018, the presence of the virus was confirmed by DAS-ELISA.

### RT-PCR Detection

To examine virus, total RNAs were isolated from young leaves excised from greenhouse shoots according to Meisel et al. (2005). cDNAs were generated from 5 μg of the total RNAs using cloned ReverAid Reverse Transcriptase (Fermentas, United States) and were subjected to PCR. The primers used for amplification of a 245-bp fragment of 3′UTR were 5′-gtc-tct-tgc-aca-aga-act-ata-acc-3′ and gta-gtg-gtc-tcg-gta-tct-atc-ata-3′. The primers used for amplification of a 442-bp fragment of *HC-Pro* gene were 5′-cca-gga-atg-agc-gga-ttt-gtg-gt-3′ and 5′-cat-gtg-aaa-att-gtg-gat-agt-tat-cca-tca-c-3′. The primers used for amplification of a 990-bp fragment of RNA-dependent RNA polymerase gene were 5′-gaa-gga-aat-ttg-aaa-gca-gtt-ggagc-3′ and 5′-cat-tca-cra-art-acc-grc-aaa-tgc-a-3′. PCR products were separated by electrophoresis on 1.2% (w/v) agarose-ethidium bromide gels.

### DAS-ELISA

The leaves of plum plants were disrupted in liquid nitrogen. Powered material was resuspended in three volumes of extraction buffer containing 50 mM Tris–HCl (pH = 8.0), 10 mM EDTA (pH = 8.0), 10% glycerol (v/v), and 30 mM 2-mercaptoethanol. The total proteins were extracted for 20 min at 25°C and then centrifuged for 10 min at room temperature and the supernatant was used for analysis. PPV infection was evaluated using Double Antibody Sandwich ELISA (DAS-ELISA) by the kit of LOÅWE Biochemica GmbH (Sauerlach, Germany). DAS-ELISA assay was applied to the leaves using rabbit polyclonal antibodies against the coat protein of PPV (Anti-Virus-IgG, Anti-Virus-IgG-AP-conjugate). Absorbance was measured with an iMark Microplate reader (Bio-Rad, United States) at 415 nm.

### Western Blot Analysis

Protein extract (25 μL) from each transgenic line was extracted as described above and separated on 12.5% SDS-PAGE and transferred onto NC membrane (Bio-Rad, United States) by tank transfer. Western blot analysis was performed using rabbit polyclonal antibodies to PPV coat protein (Anti-Virus-IgG); antibody was diluted 1:500. Anti-Virus-IgG-AP was used as secondary antibody (dilution 1:3000).

### ImmunoStrip Test

The ImmunoStrip (Agdia, United States) test was used for the detection of PPV in leaves. The analysis was carried out according to the manufacturer’s protocol.

## Results

### *Agrobacterium*-Mediated Transformation of Plum and Production of Putative Transgenic Plants

**FIGURE 1:**
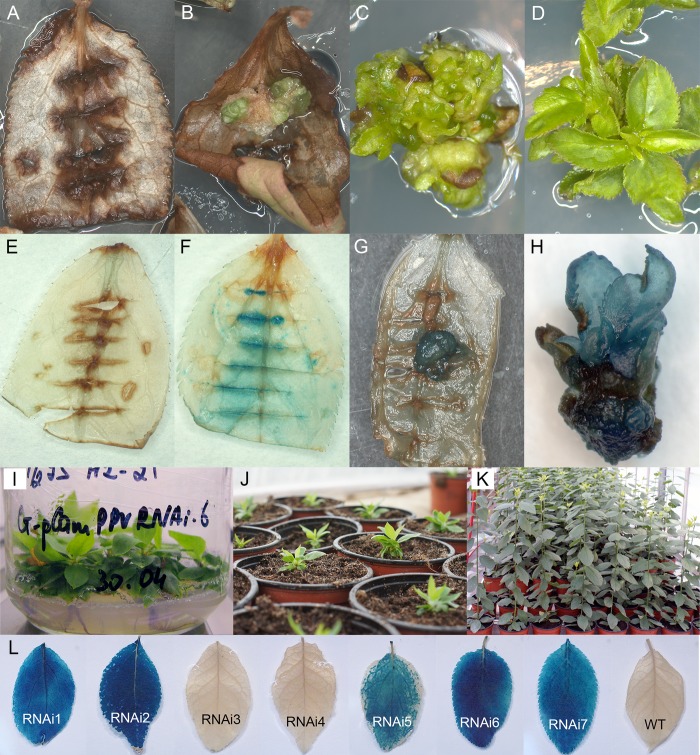
Generation and histochemical GUS analysis of putative transgenic plum plants. Wild-type **(A)** and *Agrobacterium*-inoculated leaves **(B)** on the shoot regeneration medium after 2 months of cultivation on the presence of 5 mg/L hygromycin. Differentiation of putative transformants from leaf-derived morphogenic callus on selective medium **(C)**. The proliferation of putative transformants on elongation medium supplemented with 5 mg/L hygromycin **(D)**. GUS staining of the non-transformed **(E)** and transformed **(F)** leaf explants. GUS staining of the putatively transformed callus **(G)** and the putatively transformed shoots in the cluster **(H)**. Rooting of putative transgenic plum plants rooting medium supplemented with 3 mg/L hygromycin **(I)**. The rooted plants were growing in soil **(J,K)**. Expression of *uid*A gene in leaves of independent transgenic plants **(L)**.

A total of 673 leaf explants from *in vitro* grown plum “Startovaya” were infected by *Agrobacterium* strain AGL0 containing plasmid pCamPPVRNAi to produce PPV resistant plants. A delayed selection strategy was used to generate plum transgenic plants. Three days after co-cultivation, explants were transferred onto the callus induction medium supplemented with cefotaxime to eliminate the bacteria; to stimulate the morphogenic callus induction the medium was lacking the selective antibiotic. GUS expression assay performed 2 weeks after co-cultivation, showed a faint to dark-blue coloration at the cut parts of leaf blades inoculated with bacteria (Figure 1E,F). Due to the fact that the *uidA* gene of pCamPPVRNAi construct includes an intron, blue staining was indicative of the successful transient T-DNA expression in plant cells rather than of leaked transgene expression in *Agrobacterium tumefaciens*.

After 3 weeks of culture without selective antibiotic, explants were transferred to callus induction medium supplemented with hygromycin. In 2 month of cultivation on selection medium explants became completely brown (Figure 1A). At the same time, small and green morphogenic calli started to grow at the cut edge of some explants, predominately in the middle part of the cultured leaf (Figure 1B). When some of the explants with callus pieces were tested for reporter gene expression by the histochemical assay, the callus displayed a various degree of blue coloration, indicating the activity of the reporter *uidA* gene (Figure 1G). Each developed calli was carefully removed from the leaf explant and transferred to the fresh medium for further growth and formation of morphogenic structures under selective pressure. During the next two to three subcultures on selective medium leafy-like structures were developed (Figure 1C). After a transfer into elongation medium, several of these structures had turned into the adventitious shoots (Figure 1D). Analyzed regenerated shoots displayed a clear blue staining, indicative of the stable integration and expression of the 35S::GUSintron portion of the pCamPPVRNAi vector (Figure 1H). Around 6–8 months after co-cultivation with *Agrobacterium* the regenerated plantlets were transferred to multiplication medium and then rooted in the presence of hygromycin (Figure 1I). Once a viable root system had developed, the shoots were transferred to soil (Figure 1J) and continued to grow in the greenhouse (Figure 1K).

### Molecular Analysis of Transgenic Plants

**FIGURE 2:**
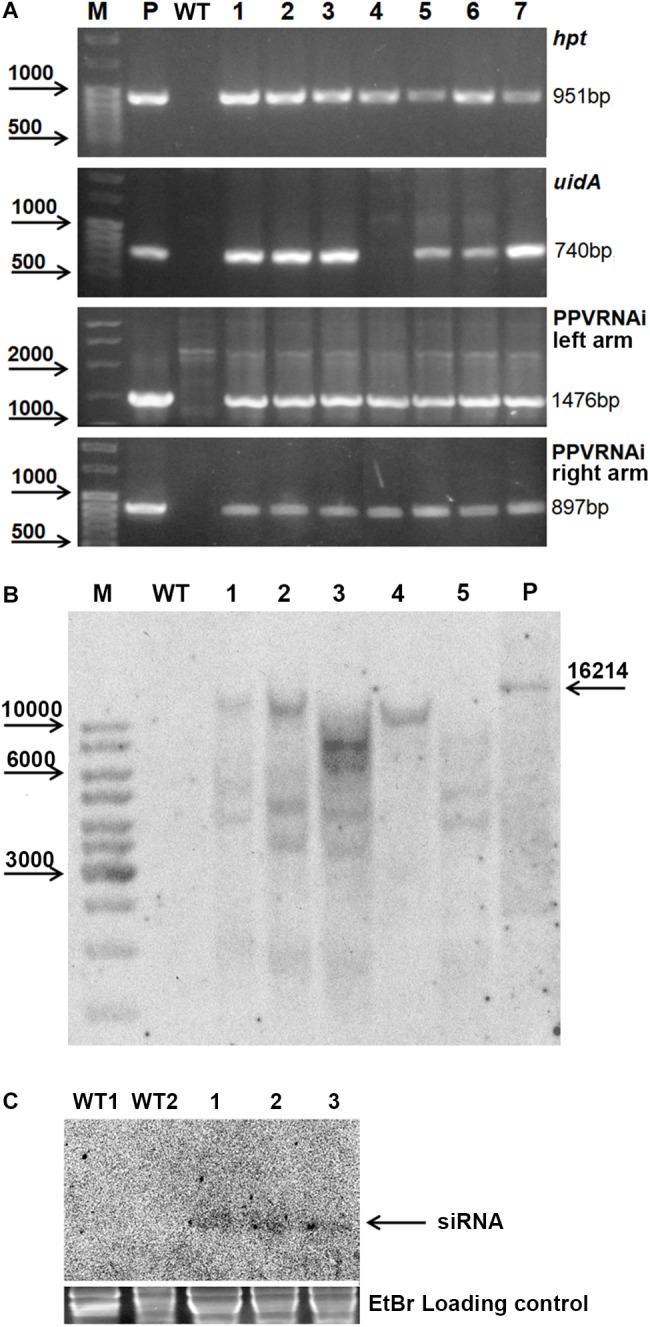
Molecular analysis of putative transgenic plum plants. PCR analysis of the putative transgenic lines **(A)**, where M – DNA marker; P – plasmid pCamPPVRNAi; WT – genomic DNA of wild-type; 1 to 7 – DNA of transgenic lines RNAi1, RNAi2, RNAi3, RNAi4, RNAi5, RNAi6, and RNAi7. Southern hybridization analysis of transgenic lines **(B)**, where M – DNA marker; WT – genomic DNA of wild-type digested with *Eco*RI; P – plasmid pCamPPVRNAi/*Xba*I; 1 to 5 – DNA of transgenic lines RNAi1, RNAi2, RNAi3, RNAi4, and RNAi6 digested with *Eco*RI. Northern Blot of transgene derived small RNAs in transgenic plum **(C)**, where WT1 and WT2 – RNA of wild-type, 1, 2, and 3 – RNA of transgenic lines RNAi2, RNAi3, and RNAi4, correspondingly.

Seven independent hygromycin-resistant lines were produced after *Agrobacterium*-mediated delivery of pCamPPVRNAi in three independent experiments (Table 1). The transformation efficiency (transgenic lines per inoculated explant) varied from 0.8 to 1.1% in various experiments (Table 1). All regenerated plants were found to be PCR positive for the introduction of selective gene *hpt*, as well as for the presence of both left and right arms of ihpRNA construct (Figure 2A). At the same time one of the transgenic lines, RNAi4, has a truncated insert of T-DNA, since the expected 740 bp fragment of reporter *uidA* gene was not correctly amplified from isolated DNA after PCR reaction (Figure 2A). The molecular results were further confirmed histochemically. Assay for β-glucuronidase activity in mature leaves of 1-year-old greenhouse-grown plants revealed no reporter gene expression in RNAi4 transgenic line (Figure 1L). Surprisingly, the silencing of the *uidA* gene was also found in tissues of transgenic line RNAi3 previously showed an amplification of the expected fragment of a reporter gene. All the other independent transgenic lines displayed a high expression level of *uidA* gene (Figure 1L) and the intensive blue stating of leaves was constantly observed during all the years of cultivation in the greenhouse.

**Table 1:** Efficiency of the genetic transformation of European plum “Startovaya” by the pCamPPVRNAi vector.

Experiment	Number of explants	Number of explants produced hygromycin resistant calli	Number of independent PCR positive lines			Transformation efficiency (%)
			Hyg+	GUS+	RNAi+	
1	452	28	5	4	5	1.1
2	119	7	1	1	1	0.8
3	102	3	1	1	1	1.0
Total	673	38	7	6	7	1.0

All transgenic plants were grown to maturity and no variation in foliar morphology and growth habit between transgenic events and non-transgenic plants of “Startovaya” was observed (Figure 1D). Since it was not possible to investigate the response against viral infection in all produced transgenic lines, five independent events (RNAi1, RNAi2, RNAi3, RNAi4, and RNAi6), both with and without *uidA* expression, were chosen for further PPV tests. To confirm the stable integration of T-DNA into the genome of selected events, Southern blot analysis was performed using a DNA probe corresponding to the sequences of the hairpin arm and octopine synthase gene terminator. All tested lines showed a positive signal, except for the non-transgenic control plant (Figure 2B). Since a restriction enzyme *Eco*RI cuts T-DNA of pCamPPVRNAi plasmid only once, one band would be expected to correspond to the number of loci carrying the transgenes. Among analyzed transgenic lines the single integration pattern was found only in RNAi4 transgenic line. Three multiple insertions were detected in the RNAi1, RNAi2, and RNAi6 lines, at least four copies of transgenes were found in line RNAi3. To ensure that transgenic plum plants constitutively express the introduced cassette and process siRNA molecules, a Northern blot analysis was performed. A low molecular weight RNAs was extracted from the leaves of the transgenic RNAi2 line displaying the reporter *uidA* gene expression and two transgenic RNAi3 and RNAi4 lines, where the *uidA* gene is silenced. The blot was hybridized with an alkaline phosphatase labeled DNA probe specific for PPV *CP* fragment (897 bp) prepared by PCR. The result showed that all analyzed transgenic plum plants produce the small interfering RNAs; no signals were detected in non-transgenic plants (Figure 2C).

### Evaluation of the Susceptibility of Transgenic Plum Lines to Artificial *Plum pox virus* Infection

Transgenic lines generated after *Agrobacterium*-mediated transformation with hp-PPV construct were artificially inoculated with PPV in the spring of 2009 by grafting of infected bud.

#### 2010

**FIGURE 3:**
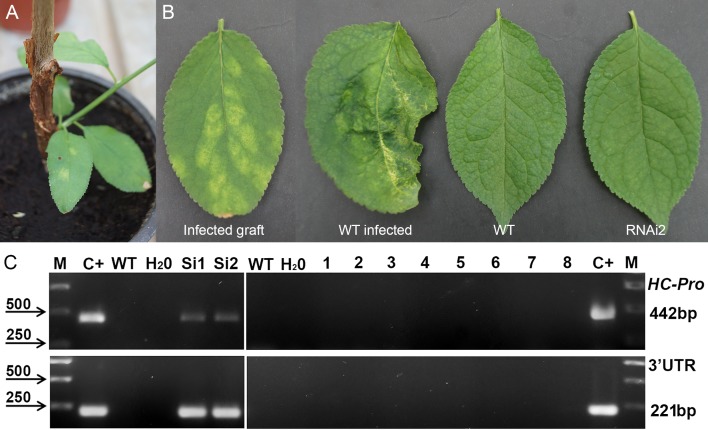
Analysis of infected transgenic plum plants next year after inoculation with PPV. Inoculation on the transgenic plant through bud grafting **(A)**. Leaf symptoms caused by *Plum pox virus* **(B)**. RT-PCR assay of transformed plum plants infected by PPV **(C)**, where M – DNA marker; C+ –infected grafting; WT – uninfected wild-type; H_2_O – water; Si1 and Si2 – infected wild-type; 1 to 8 – transgenic individuals from RNAi1-1, RNAi1-2, RNAi2-1, RNAi2-2, RNAi3-1, RNAi3-2, RNAi4, and RNAi6. Amplification of 442 bp fragment of PPV *HC-Pro* gene and 245 bp fragment of 3′UTR PPV sequence.

Next year after cold-induced dormancy virus-containing buds that were grafted on the WT and transgenic plants developed into small branches with obvious PPV symptoms on leaves (Figure 3A). In average, branches were produced from a half of the infected buds, and there were no significant differences between transgenic and control plants in grafts survival. Fifteen months post-grafting no visible symptoms of virus infection were observed on leaves of tested transgenic plum plants (Figure 3B). At the same time, typical yellow diffuse spots and rings have appeared on leaves of non-transgenic “Startovaya” plants grafted with PPV-infected buds (Figure 3B). The RT-PCR test performed in June of 2010 confirmed the presence of the virus in total RNA extract of WT grafted plants (Figure 3C). All evaluated individual transgenic plants infected with virus-containing buds were RT-PCR negative for the presence of viral 3′UTR sequences and B-gene of HC-Pro protease (Figure 3C and Table 2).

**Table 2:** *Plum pox virus* detection in transgenic plum plants.

Lines/year of assessment	2010		2014			2018	
	
	Visual symptoms^∗^	RT-PCR	Visual symptoms^∗^	RT-PCR	Western blot/ ELISA/ImmunoStrip	Visual symptoms^∗∗^	ELISA
WT	−	−	−	−	−	−	−
WT infected	+	+	+	+	+	+	+
Graft on WT	+	+	+	+	+	n.a.	n.a.
RNAi1	−	−	−	−	−	−	−
Graft on RNAi1	+	n.d.	+	+	+	n.a.	n.a.
RNAi2	−	−	−	−	−	−	−
Graft on RNAi2	+	n.d.	+	+	+	+	+
RNAi3	−	−	−	−	−	−	−
Graft on RNAi3	+	n.d.	n.a.	n.a.	n.a.	n.a.	n.a.
RNAi4	−	−	−	−	−	−	−
Graft on RNAi4	+	n.d.	n.a.	n.a.	n.a.	n.a.	n.a.
RNAi6	−	−	−	−	−	−	−
Graft on RNAi6	+	n.d.	+	+	+	n.a.	n.a.

*n.d. – not determined; n.a. – not available (infected grafts died off by the time of evaluation); ^∗^eight to ten individual transgenic plants for each line and WT were evaluated for symptoms; ^∗∗^two to three individual transgenic trees for each line and WT were evaluated for symptoms.*

#### 2014

**FIGURE 4:**
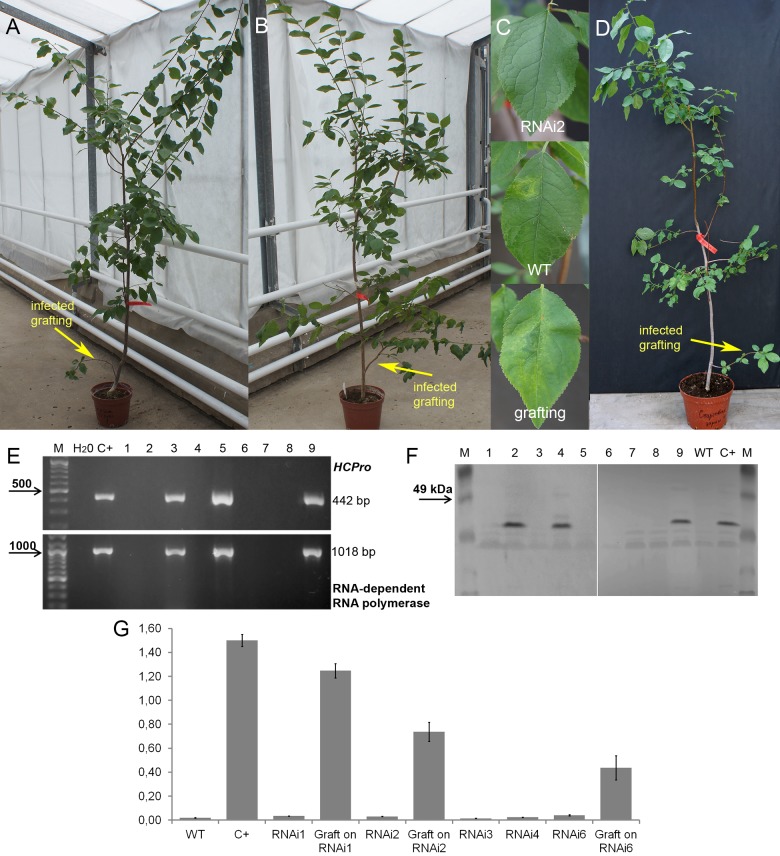
Analysis of infected transgenic plum plants after 5 years from inoculation. Transgenic plum RNAi2 **(A)** and RNAi6 trees **(B)** with infected graft formed branches. Leaf symptoms caused by *Plum pox virus* **(C)** in transgenic lines RNAi2, infected wild-type (WT) and infected grafting. Infected wild-type with infected graft formed branches **(D)**. RT-PCR assay of transformed plum plants infected by PPV **(E)**, where M – DNA marker; H_2_O – water; C+ – infected wild-type; 1 – uninfected wild-type; 2 – RNAi1; 3 – infected graft on RNAi1; 4 – RNAi2; 5 – infected graft on RNAi2; 6 – RNAi3; 7 – RNAi4; 8 – RNAi6; 9 – infected graft on RNAi6. Western blot analysis of transgenic plum plants **(F)**, where M – marker; C+ – infected wild-type; WT – uninfected wild-type; 1 – RNAi1; 2 – infected graft on RNAi1; 3 – RNAi2; 4 – infected graft on RNAi2; 5 – RNAi3-1; 6 – RNAi3-2; 7 – RNAi4; 8 – RNAi6; 9 – infected graft on RNAi6. DAS-ELISA assay was applied to the leaves of infected plum plants **(G)**, where WT – uninfected wild-type; C+ – infected wild-type.

Plum plants artificially inoculated with PPV by grafting were grown in greenhouses and developed into trees during the subsequent four seasons (Figure 4A,B,D). Due to regulatory and quarantine restrictions associated with the GM nature of plants and persistent PPV infection, it was impossible to maintain a number of grafted plants in the greenhouse. Two to three trees of each transgenic line were maintained. All the seasons we monitored the appearances of the virus symptoms on transgenic and non-transgenic plants. After 5 years of cultivation, PPV symptomatic leaves were still not found at any of transgenic trees grafted with virus-infected buds (Figure 4A–C and Table 2). This visual observation was confirmed by various tests including RT-PCR (Figure 4E), Western blot (Figure 4F), DAS-ELISA (Figure 4G), and ImmunoStrips tests (Supplementary Figure S1). Important to note that 5 years after grafting, trees of three independent events, RNAi1, RNAi2, and RNAi6, still carried the survived infected branches derived from virus-containing grafted buds (Figure 4A,B). The survived branches displayed clear foliar symptoms of Sharka disease (Figure 4C) and were RT-PCR and ELISA positive. The presence of PPV coat protein in extracts of symptom-expressing leaves was also detected by Western blot and ImmunoStrips (Table 2). Observation indicated that these branches, being a part of a tree, constantly infected transgenic plants with PPV through the vascular tissue all 5 years of cultivation.

#### 2018

**FIGURE 5:**
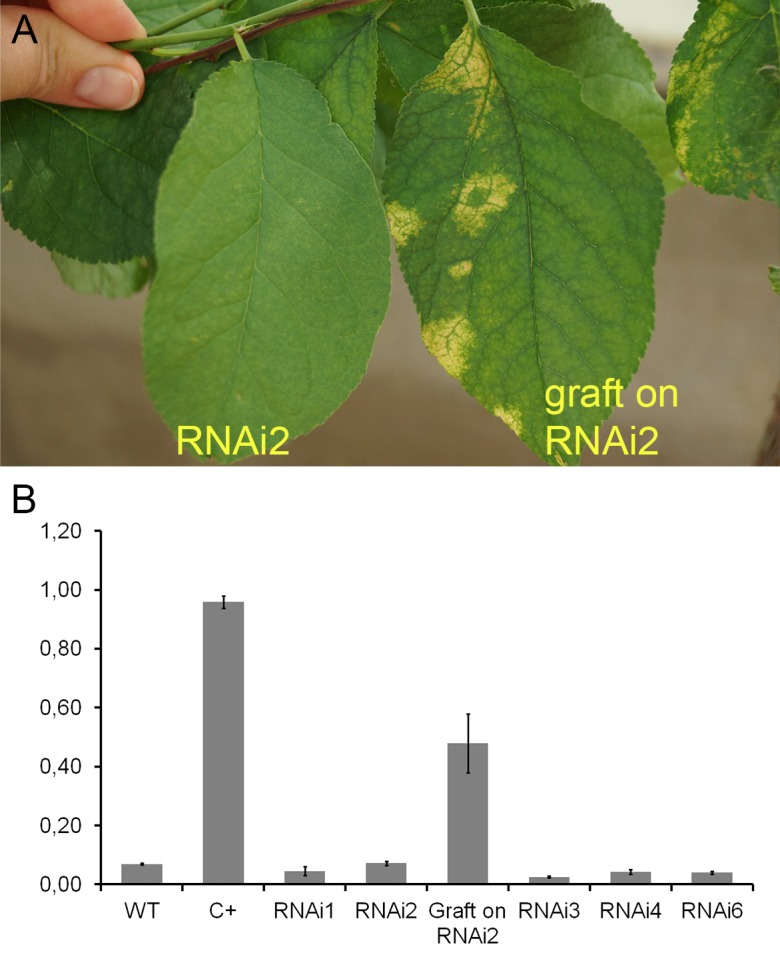
Analysis of infected transgenic plum plants after 9 years from inoculation. Leaf symptoms caused by *Plum pox virus* **(A)**. DAS-ELISA assay was applied to the leaves **(B)**, where WT – uninfected wild-type; C+ – infected wild-type.

During the 2015–2017 most of the infected branches were died off on the transgenic trees. By year nine of post-grafting, only one transgenic event, RNAi2, still carried the infected graft as an active branch. During the vegetation periods of 2018, the leaves of the survived graft had typical viral symptoms, while the remainder of the tree (= transgenic RNAi2) showed no incidence of Sharka disease (Figure 5A). ELISA assays confirmed PPV presence in 9-year-old graft; the tissues of the transgenic RNAi2 tree were ELISA negative (Figure 5B). WT “Startovaya,” challenged by a virus at the same time as the transgenic RNAi2 event, also was ELISA positive and displayed the viral symptoms on leaves. Unlike RNAi2, the infected grafts on non-transgenic “Startovaya” plants withered several years after inoculation. The trees of other independent transgenic events, infected by the same method, lost infected grafts during 2013–2017, but unlike the WT “Startovaya,” transgenic RNAi1, RNAi3, RNAi4, and RNAi6 showed effective resistance to PPV, because there were no symptomatic leaves after 9 years of cultivation (Table 2).

## Discussion

Evidence from the previous reports and the present study indicate that the examination for PPV resistance in transgenic fruit trees depends on a number of biological, environmental and social factors (Cirilli et al., 2016). Over the years, there have been several attempts to evaluate virus resistance in transgenic plum and other stone fruit species under various *in vitro* and *ex vitro* conditions (Prieto, 2011; Monticelli et al., 2012; Song et al., 2013; Ravelonandro et al., 2014; Zhao and Song, 2014; García-Almodóvar et al., 2015; Polák et al., 2017) In conventional breeding, two to four growing seasons are recommended to analyze the individual genotype, including symptom score, polymerase chain reaction (PCR) and serological (ELISA) analysis (Cirilli et al., 2016). The variability of the PPV isolates, the method of infection, delayed responses to inoculation, seasonal effects and environmental conditions are among the key factors affecting the assessment in both GM and conventional germplasms (Garcia et al., 2014; Rimbaud et al., 2015; Cirilli et al., 2016). The transgenic nature of the plants is an additional important issue for successful evaluation. In various countries, the appropriate permits are required for conducting GM field trials. In such circumstances, the evaluation is mainly performed in greenhouses certified to grow GM plants. Taking into account the quarantine status of PPV in Russia, the greenhouse approach was also applied in the present study.

To date, the assessment of transgenic plum expressing the hairpin-RNA constructs mainly lasted from 2 to 4 years in greenhouse-controlled condition (Wang et al., 2009; Prieto, 2011; Ravelonandro et al., 2014; García-Almodóvar et al., 2015). Most research on transgenic virus-resistant *Prunus* species was conducted on young plants, and only a few field trials have focused on the evaluation of adult transgenic trees. Here we report the analysis of mature trees, which were monitored in protected greenhouses up to 9 years. This, however, required an extended space to grow trees in pots and additional costs to provide the artificial seasonal changes. For this reason, we have to limit the number of evaluating transgenic trees for each independent event from 8 to 10 at the beginning till two trees at the end of the evaluation.

In our study, the self-rooted plum plants were challenged with the Russian PPV-M isolates [PS (AJ243957)] that belongs to PPV-M serotype. Naturally infects *Prunus* molecularly similar PPV-M, for most outbreaks of Sharka disease in industrial plum and peach plantations. Disease delivery into 1-year-old transgenic plants was performed by the artificial virus inoculation by bud grafting. According to various reports, mechanical inoculation provides a much higher infection pressure than the inoculation with vectors such as aphids (Malinowski et al., 2006). This, in turn, ensures a deeper screen for virus resistance of individual events. For example, Malinowski et al. (2006) reported that field grown transgenic trees of C-5 remained always healthy throughout the 8-year study when plants were challenged with a natural aphid inoculation. In the case of the artificial chip bud inoculation with the same virus strain, host transgenic C-5 plants found to displayed minor diseases symptoms up to several years of evaluation (Malinowski et al., 2006).

The results of our work confirmed the efficiency of direct viral delivery into the plum plants. Next year after inoculation clear symptoms of Sharka disease were detected in leaves of non-transgenic “Startovaya” plants (Figure 3B). The visual symptoms completely corresponded to RT-PCR analysis for the virus accumulation (Figure 3C). In contrast, none of five independent transgenic events expressing the PPV *CP* hairpin construct showed a positive reaction upon virus inoculation. A healthy phenotype was still observed in subsequent years when the grafted buds developed into the infected branches. By the fifth year of the challenge experiment, the transgenic events showed no accumulation of the virus to a level detectable by RT-PCR, Western blot, ELISA or ImmunoStrips (Figure 4E–G and Supplementary Figure S1), while the dose of viral inoculum was constantly high due to a connection of host trees with a vascular tissue of growing infected branch (Figure 4A,B). This result is contrasting to the results of recently published reports of Polák et al. (2017), who could find the mild symptoms on leaves of transgenic C-5 trees 5 years post inoculation. Moreover, the accumulation of PPV in transgenic C-5 trees was detected by RT-PCR and DAS-ELISA. It should be noted, however, that in the study of Polák et al. (2017), transgenic C-5 was directly grafted as a scion onto a peach rootstock infected with PPV-rec isolate, which is highly homological, but rather different from PPV-M used in our study.

By the fifth and ninth years after initial inoculation, the OD values of ELISA test of the WT and infected transgenic plants of “Startovaya” were very low (Figure 4G, 5A). In contrast, the OD of infected WT increased up to 50–75 times and was stably high including the latest year of evaluation. The type and intensity of symptoms were confirmed by the molecular analysis using primers designed for the detection of viral RNA fragments of PPV HC-Pro and RNA-dependant RNA polymerase (Figure 4E) and also by Western blot assay (Figure 4F) carried out to prove the presence of PPV *CP*. It was also obvious that infected branches growing on the transgenic trees were not recovered from viral symptoms. ELISA tests conducted 5 and 9 years after inoculation found that the OD of the extracts from infected branches was 1.5–2 times lower than the OD of the infected WT. Similarly, the intensity of the test line of ImmunoStrip (Supplementary Figure S1) was less bright but still obvious to confirm the virus persistence in infected grafts, continuing to grow on transgenic RNAi1 and RNAi6 trees. We could not associate the reduction of virus accumulation level in infected graft with the delivery effect of siRNA from cells of the transgenic host since the overwhelming majority of infected branches died off within the 3–6 year of the experiment.

The inability of infected WT branches to cure PPV infection after long time conjugation with transgenic plum tissues contradicts the idea of trans-grafting to meet the biosafety requirements for the fruit production (Lemgo et al., 2013). Trans-grafting is referring to the combination of non-transgenic scion and transgenic rootstock, whereby the viral sustainability of the scion will be ensured by the movement of resistance signal through the vascular system from rootstock to non-transgenic part of the tree (Limera et al., 2017). In the recent study conducted on the trans-grafted *Prunus* trees consisted of transgenic cherry rootstock (*P. cerasus* × *P. canescens*) and non-transgenic scion of sweet cherry (*P. avium* L.), the transfer of siRNAs from rootstock expressing the hpRNA construct was proved to provide the resistance to the *Prunus necrotic ringspot virus* (Zhao and Song, 2014). Similarly, transgenic siRNAs (derived from *IPT* and *IAAM* hairpins produced in the transgenic rootstock) were found to cross the graft junction in walnut, but accumulation was very low and observed only in kernels (Haroldsen et al., 2012) It is noteworthy that in the same report, siRNAs mobility across the graft was not confirmed for herbaceous tomato species and the trans-grafted plants did not accumulate a detectable amount of siRNAs derived from *IPT* and *IAAM* hairpins.

Since in our study the infected grafted buds/branches were developed together with the transgenic host and located below transgenic branches, the host plant would be considered as a rootstock to some extent. From this point of view, there was no success in the provision of resistance signal into infected branches, as they displayed evident Sharka symptoms year by year, slowly decayed and eventually died off. In another report, the lack of transportation of hpRNA-mediated *uidA* gene silencing signals from transgenic rootstocks to a scion overexpressing the reporter gene was previously reported in greenhouse grown apple plants (Flachowsky et al., 2012). Such apparently contradictory results indicate that transition of various silencing signals from transgenic rootstocks expressing RNAi-eliciting constructs to non-transformed scions is a complex process depending on plant species and target sequences. It was also shown that the various environmental factors (temperature, light intensity, and humidity) significantly affect the systemic movement of silencing signal in herbaceous species to achieve RNAi-mediated virus resistance (Patil and Fauquet, 2015). More investigations are required in plum to clarify the practical implication for transgrafting technology aimed to produce virus-resistant scions grafted on GM rootstocks.

In recent years to overcome laborious long-term evaluation in greenhouse and field trials, more attention has been paid to methods involved *in vitro* grafting procedures (Lansac et al., 2005; Monticelli et al., 2012; García-Almodóvar et al., 2015). This approach involves the grafting of *in vitro* transgenic apexes onto the shoots of peach rootstock “GF305” known for its high susceptibility to PPV. In the recent report of García-Almodóvar et al., 2015 the results of *in vitro* tests of various transgenic plum events matched with 100% accuracy with the greenhouse assessments. *In vitro* evaluation for virus resistance prior to detailed assessment in the greenhouse would definitively help to identify the promising lines at early stages. It should, however, notice that this methodology was not yet tested on many rootstock-scion combinations, and certain issues with the effectiveness of *in vitro* grafting for other plum genotypes should be considered. Another drawback of this method is the absence of clear PPV symptoms in both *in vitro* parts of rootstock and scion. Therefore, it is necessary to carry out molecular or serological tests in order to reveal a preliminary level of resistance.

Despite enormous potential, *in vitro* and *ex vitro* procedures were mainly used in plum as “proof-of-concept” studies to determine whether one or another strategy can be further used to obtain viral resistant mature trees. To date, “HoneySweet” (formerly C-5) is still the only one transgenic plum event, which was deeply examined under both greenhouse and field conditions for the stability of engineered PPV resistance over the years. A group of scientists from United States, Poland, Czechia, Romania, and Spain performed numerous comparative assessments for the biological, serological, and molecular characteristics of transgenic C-5 trees (Malinowski et al., 2006; Scorza et al., 2013; Polák et al., 2017). “HoneySweet” is the first perennial tree fruit deregulated for commercial production in United States (Scorza et al., 2013). Various field tests proved that “HoneySweet” trees, which were challenged by aphid-transmitted PPV, were stably uninfected under the natural environment. At the same time, transgenic trees were not able to completely withstand to movement and multiplication of virus when the direct ingress of PPV by graft-inoculation was performed or trees were grown on PPV-infected rootstock. Mild PPV symptoms on leaves of some trees could be observed for both short and long period of cultivation (Malinowski et al., 2006). Although symptoms disappeared gradually year by year, it was required several years to completely recover infected “HoneySweet” trees from Sharka disease (Polák et al., 2017). Although the graft-inoculation is not the natural mode of PPV infection, the latently infected trees could be a potent source for the aphid-vectored dispersal into non-resistant genotypes.

Another shortcoming of the most studies involved transgenic plum plants expressing antiviral sequences is the absence of information on pomological traits affected by PPV infection. On the one hand, it’s quite difficult to produce plum fruits in a greenhouse during a short assessment period because plants do not reach the reproductive stage. On the other hand, due to the regeneration from seed derived tissue, independent events may exhibit large differences in pomological traits related to a new genetic mix, not with a viral effect. The only exception is field evaluated mature trees of “HoneySweet.” After 11 years of cultivation in the Czechia, GM trees displayed a minimal change in the fruit quality after viral inoculation (Sochor et al., 2015; Krška et al., 2017).

Despite the prolonged cultivation time in the present study, we also failed to produce fruits from greenhouse grown transgenic plum plants because “Startovaya” is not self-pollinated variety, so its fruit formation largely depends on compatibly flowering pollinator. To produce fruits and conduct a comparative analysis of morphological and pomological traits of transgenic plum trees under orchard circumstances, a field trial of GM plum was established in 2012 under official permit within the isolated area owning by the Russian Institute of Horticultural Crops Breeding. Unfortunately, due to an issue of a new moratorium for GM plants cultivation in Russia in 2016, the field test renewal was not granted and experimental plum trees were destroyed. This encourages us to produce transgenic plum fruits by an artificial pollination of greenhouse-grown trees and we hope to get results in the nearest future.

Long-time transgene-derived immunity through expression of ihpRNAs, as shown here, would likely protect our plum trees against natural and artificial inoculations with a different homologically close PPV strains. In the present study plum plants expressing ihpRNAs construct containing conserved genomic regions from PPV-D, were successfully withstand to inoculation with a harmful PPV-M isolate. Our data is perfectly matched with previous results indicated that strong expression of ihpRNAs from conserved sequences of PPV-D and -M strains provides the resistance to inoculation with viral isolates belonging to various taxonomic groups, including D, M, Rec, C, and EA strains (Ravelonandro et al., 2014). Moreover, siRNAs from PPV-D CP hairpins have ensured the resistance to the combination of the PPV-rec isolate with other fruit tree viruses frequently associated with PPV infection in the natural environment, such as *Prune dwarf virus* (PDV) and *Apple chlorotic leaf spot virus* (ACLSV) (Polák et al., 2017).

In recent years, the noteworthy achievements and the future directions to improve various agronomical and consumer traits of plum by the innovative technologies, such as genome modification and genome editing were reviewed (Ilardi and Tavazza, 2015; Petri et al., 2018). However, all these modern approaches are broken down by the impossibly to introduce favorable changes to the existing varieties without destroying their original characteristics. Among the major industrial fruit trees, plum is probably the significantly lagging species in development and application of genetic transformation protocols based on the morphogenic response of somatic tissues. In contrast to plum, significant progress is achieved in the regeneration of adventitious transgenic shoots from adult/somatic tissues of various fruit trees including apple (Malnoy et al., 2010), pear (Matsuda et al., 2005), fig (Yancheva et al., 2005), clementine (Cervera et al., 2008), citrus (Orbović et al., 2015), sweet orange (Zanek et al., 2008). No doubt, the experimental transgenic plum plants generated from seed-derived tissues should be largely involved in the plum enhancement by conventional breeding and for functional plum genomic. Some recent data indicate that PPV resistance obtained in seed-derived transgenic plum events is inherited in next seed generations (Scorza et al., 2016). Nevertheless, the incorporation of transgenic traits into commercial varieties by hybridization is still time- and cost-consuming procedure, even with the use of rapid breeding technologies like “FastTrack” (Petri et al., 2018).

The presented study is a significant step in engineering commercial varieties of plum for PPV resistance and other viable traits. Clonal sources of our transgenic shoots regenerated from adult plant material prompt to apply the described transformation procedure for conferring a broader range of transgenic characteristics to valuable cultivars. In the present study the transformation efficiency of 1% is generally fit to 0.3–5.6% efficacy of genetic transformation described for leaf explants of apricot (Petri et al., 2015), almonds (Ramesh et al., 2006), cherry (Song, 2015), and shoot tips of peach (Sabbadini et al., 2015).

The usefulness of described here transformation protocol was confirmed by our recent study (Sidorova et al., 2018). By means of *Agrobacterium*-mediated transformation procedure using *in vitro* leaf explants we successfully generated transgenic events of Russian clonal plum rootstock “Elita” [(*Prunus pumila* L. × *P. salicina* Lindl.) × (*P. cerasifera* Ehrh.)]. The preliminary results of 2-year evaluation confirmed the efficiency of described here hairpin constructs for protecting GM rootstock from virus attack in greenhouse-controlled conditions. Taking into account the resistance data presented here, we hope to establish in near future mature plum trees consisted of PPV resistant scion grafted on PPV resistant rootstock.

## Data Availability

The datasets generated for this study can be found in gossort, http://reestr.gossort.com/reestr/sort/9908465.

## Author Contributions

TS, RM, and SD conceived and designed the experiments. TS, RM, and AP performed the experiments. TS, DM, AP, and SD analyzed the data. SD contributed to reagents and materials. TS and DM wrote the manuscript with assistance from all authors.

## Conflict of Interest Statement

The authors declare that the research was conducted in the absence of any commercial or financial relationships that could be construed as a potential conflict of interest.

## Footnotes

1 http://reestr.gossort.com/reestr/sort/9908465
