# Supplementary material for: Agrobacterium-Mediated Transformation of Russian Commercial Plum cv. “Startovaya” (Prunus domestica L.) With Virus-Derived Hairpin RNA Construct Confers Durable Resistance to PPV Infection in Mature Plants
Source: Front Plant Sci. 2019 Mar 12;10:286. doi: 10.3389/fpls.2019.00286 (PMC6423057; doi:10.3389/fpls.2019.00286)
Supplement: Supplementary file 1 [file Image_1.pdf]

Supplementary Material

Agrobacterium-mediated transformation of Russian commercial plum cv. 'Startovaya' (*Prunus domestica* L.) with virus-derived hairpin RNA construct confers durable resistance to PPV infection in mature plants

Tatiana Sidorova*, Roman Mikhailov, Alexander Pushin, Dmitry Miroshnichenko, Sergey Dolgov

*** Correspondence: Corresponding Author: sidorovat@rambler.ru**

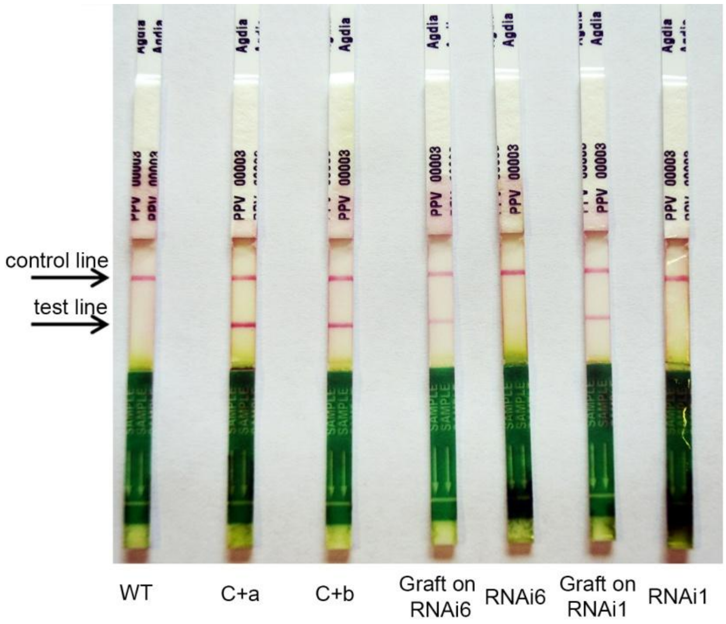

Supplementary Figure 1. Immunostrip assays to determine the virus accumulation in the inoculated plum plants. The lower line reveals positive reaction at the protein level (PPV coat protein accumulation). WT – non-transgenic uninfected wild-type plant, C+a and C+b – two individuals of non-transgenic infected by grafting wild-type trees, RNAi1 and RNAi6 – independent transgenic events inoculated with a virus; grafts on RNAi1 and RNAi6 – branches developed on host transgenic trees from grafted infected buds. The test is carried out using the leaf extracts after 5 years of the inoculation.
